# Encouraging HPV Vaccination via an Evolutionary Theoretical Approach: A Randomized Controlled Study in Japan

**DOI:** 10.3390/vaccines10050701

**Published:** 2022-04-29

**Authors:** Tsuyoshi Okuhara, Hiroko Okada, Eiko Goto, Aiko Tsunezumi, Yumi Kagawa, Takahiro Kiuchi

**Affiliations:** Department of Health Communication, School of Public Health, The University of Tokyo, 7-3-1 Hongo, Bunkyo-ku, Tokyo 113-8655, Japan; okadahiroko-tky@umin.ac.jp (H.O.); testgotou1982@gmail.com (E.G.); ahtsunez@m.u-tokyo.ac.jp (A.T.); yumi.kagawa@gmail.com (Y.K.); tak-kiuchi@umin.ac.jp (T.K.)

**Keywords:** HPV vaccination, HPV vaccines, vaccine hesitancy, cervical cancer, evolutionary psychology, health communication

## Abstract

In recent years, researchers have discussed the introduction of an evolutionary perspective into public health and health behavior research. We aimed to examine the effects of messages that target the fundamental human motive of kin care on HPV vaccination recommendations among mothers with daughters, based on an evolutionary theoretical approach. This study consisted of a three-arm parallel-group single-blinded randomized controlled study. A web-based survey was conducted from 7 to 8 October 2021 in Japan. Mothers with daughters (*n* = 969) were randomly assigned either to a group that received an intervention message that targeted the fundamental motive of kin care, or that targeted the fundamental motive of disease avoidance, or a control message. Intention to have daughter(s) receive HPV vaccination was assessed both before and right after reading the messages. A one-way ANOVA with Tukey’s or Games–Howell test was conducted. An intervention message targeting the fundamental motive of kin care and disease avoidance significantly increased intention of vaccination versus a control message (*p* < 0.001, respectively). There was no significant difference between the two intervention groups. The evolutionary theoretical approach that focuses on fundamental human motives may have the potential to extend the communication strategy for HPV vaccination recommendations. Health professionals may be recommended to deliver messages that target the fundamental motive of kin care as well as messages about the susceptibility and severity of cervical cancer and vaccine efficacy (e.g., “Getting cervical cancer can prevent childbirth. To protect your daughter and your future grandchildren, get your daughter vaccinated against HPV”). However, the present study only evaluated HPV vaccination intentions in Japanese mothers with daughters. Future studies should evaluate vaccination behavior in a wider range of subjects to confirm that the evolutionary theoretical approach promotes HPV vaccination.

## 1. Introduction

Cervical cancer is the fourth most common cancer in women, with over 570,000 new cases a year [1]. Cervical cancer is a preventable disease, with several strategies for prevention and treatment, but it is mostly prevented through human papillomavirus (HPV) vaccination efforts. World Health Organization announced the “Global strategy to accelerate the elimination of cervical cancer as a public health problem” in November 2020 [1]. It has suggested vaccinating 90 percent of girls against multiple strains of HPV by 15 years of age [1]. However, although higher income countries tend to achieve higher vaccine coverage, coverage remains low in some high-income countries such as 46% in the U.S. and 31% in Germany (target population who received the last dose of HPV vaccine in 2018) [2]. A recent study estimated the final dose of HPV vaccine coverage in 2019 for females remained at only 40% in high income countries, and much lower in other countries [3].

The situation is critical especially in Japan. Following the revision of the Japanese immunization law in April 2013, HPV vaccines were positioned in “Routine A” of the national immunization program [4]. Although the vaccines in Routine A are not mandatory, the local governments provide individual recommendations known as “proactive recommendations”. Girls in the sixth year of elementary school to the first year of high school who wish to receive the HPV vaccines can do so free of charge at a local clinic because the vaccination costs are covered by public funds. However, proactive recommendation of HPV vaccination was suspended by the Ministry of Health, Labour and Welfare in June 2013 in Japan after negative campaigns by mass media about severe adverse reactions allegedly caused by HPV vaccination [5]. Notifications and vaccination coupons to encourage vaccination were no longer sent to girls of eligible ages due to the suspension. Since then, the HPV vaccination rate among age-eligible girls has been stagnating, with less than one percent of girls being vaccinated [2]. Vaccine hesitancy—the reluctance or refusal to vaccinate despite the availability of vaccines—has been listed by the WHO as one of the top 10 global health threats and is also a critical issue in HPV vaccination [6,7].

Various models and theories have been used in studies of methods to encourage HPV vaccination, such as the health belief model, protection motivation theory, and theory of planned behavior [8,9,10]. These models and theories emphasize cognitive beliefs about health behaviors, such as perceived susceptibility of infection and perceived severity of infection. However, the effects of behavioral change resulting from interventions using these theories and models are not as large as has been expected [11]. Existing cognitive behavioral models have been criticized for focusing on proximate causes of cognitive influence on health behaviors at the expense of ultimate causes of human behaviors [12].

In the present study, we adopt another approach grounded in an evolutionary theoretical framework by focusing on fundamental human motives. In recent years, researchers have discussed the introduction of an evolutionary perspective into health behavior research [13,14], and The Lancet published an evolutionary public health series in 2017 [15,16,17]. Those studies suggest that, in order to understand health behavior, we must connect the various choices we make in our day-to-day lives with their evolutionary meaning. Evolutionary biologists have presumed that all living organisms have been selected to maximize their relative success at passing genes on to future generations via either direct reproduction or helping kin reproduce, which they call inclusive fitness [18].

Because humans are a highly social species, they have faced and solved crucial social challenges to enhance their inclusive fitness. Evolutionary psychologists have assumed that these evolutionary challenges include self-protection (protecting oneself from enemies and predators), disease avoidance (avoiding infection and disease), affiliation (forming and maintaining cooperative alliances), status (gaining and maintaining respect and prestige of their fellow members), mate acquisition (successfully attracting and acquiring a romantic partner), mate retention (fostering long-term mating bond with that person), and kin care (investing in and caring for family and kin) [12,19,20]. These seven are the fundamental human motives to enhance inclusive fitness [12,19,20]. The humans who succeeded in solving these critical challenges successfully enhanced their inclusive fitness and became our ancestors.

According to the concept of domain specificity, which is one of the key features of modern evolutionary approaches [21,22], a different psychological system guides each decision, depending on which fundamental motive is currently paramount on an individual’s mind [12,19,20]. A fundamental motive can be activated and become paramount by external or internal cues that indicate threats or opportunities related to a specific evolutionally challenge [12,19,23]. Each of the seven fundamental motives is assumed to develop in stages as an individual grows through childhood and adolescence to old age, based on the life history theory [24]; even as later-developing motives become paramount, earlier-developed motives are ready to be active whenever pertinent threats or opportunities are perceived to arise [12,19,20]. For example, in the mature life stage, the fundamental motives of mate retention and kin care are paramount and guide decisions (e.g., when one becomes a parent, the care of one’s child becomes a top priority). Therefore, it is important to presume an audience’s paramount fundamental motive and target it when encouraging health behaviors, including vaccination.

Previous studies have shown that vaccination recommendation messages have primarily communicated the causes of infectious diseases and the benefits of vaccines [25,26,27]. Namely, communication to encourage vaccination to date has often targeted the fundamental motive of disease avoidance (e.g., “Vaccination can prevent the onset of infectious diseases”). However, in a safe and hygienic environment such as that of a modern industrialized country, the fundamental motive of disease avoidance may be inert. Additionally, prolonged exposure to similarly-themed messages generates psychological reactance and disengagement toward incoming messages, leading to ineffective persuasive outcomes [28,29].

Contrarily, the fundamental motive of kin care is paramount in the minds of people with daughters, although messages targeting this fundamental motive (e.g., Let’s have your daughter receive HPV vaccination because she may not be able to have a child if she gets cervical cancer.) have rarely been used in communication to encourage vaccination to date [25,26,30]. Therefore, we hypothesize that an HPV vaccination recommendation message that targets the fundamental motive of kin care will be equally effective or more effective in encouraging people to have their daughter(s) receive HPV vaccination than a message that targets the fundamental motive of disease avoidance.

However, to our knowledge, no study has focused on the fundamental human motives based on the evolutionary theoretical approach and examined their influence on health decision-making including HPV vaccination. The aim of this study is to examine the persuasive effects of messages that target the fundamental motive of kin care on HPV vaccination recommendations among participants with daughter(s), and to investigate the usefulness of developing messages to encourage HPV vaccination based on an evolutionary theoretical approach.

## 2. Materials and Methods

### 2.1. Intervention Program Theory

The present study is based on the theoretical framework of evolutionary psychology and hypothesizes that a message that appeals to the fundamental human motive of kin care will increase mothers’ intentions to have their daughters vaccinated against HPV in order to protect their fertility. A key component of the intervention is an intervention message that appeals to the fundamental motive. It is assumed that the intervention message will increase the participants’ corresponding fundamental motive, and that the increased fundamental motive will influence their vaccination intentions.

### 2.2. Participants and Design

**Figure 1:**
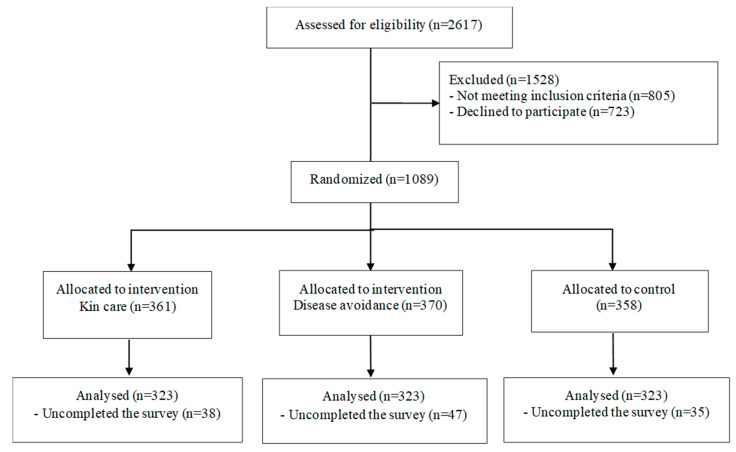
Participant Flow.

This study consisted of a three-arm parallel-group single-blinded randomized controlled study. The participants were randomly allocated 1:1:1 either to two intervention groups or a control group. A web-based survey was conducted from 7 to 8 October 2021 in Japan. Because mothers have a strong influence on their daughters’ HPV vaccination in Japan [31], mothers with daughters were recruited from people registered in a survey company database in Japan (Rakuten Insight, Inc., Tokyo, Japan). E-mails were sent to registered users who responded to screening questions. The inclusion criteria were mothers with daughter(s) in the sixth year of elementary school to the first year of high school, which corresponds to the age group eligible for HPV vaccination based on the Immunization Law in Japan. Mothers of daughters who have had at least one dose of the HPV vaccines were excluded. Figure 1 shows the participant flow diagram.

Recipients who were eligible and consented to participate were invited to complete a web-based survey. When participants consented to participate in the study via the website, they were blinded and randomly assigned to a group that received either an intervention message that targeted the fundamental motive of kin care, an intervention message that targeted the fundamental motive of disease avoidance, or a control message, using a computer-generated sequence included in the survey program.

Participants responded to questions of outcome measures and sociodemographic information. Then, participants were invited to read the intervention or control messages. Immediately after reading the messages, participants responded to questions of outcome measures again, and to a question of a manipulation check. A total of 1089 participants were randomized and 969 participants completed the survey. Tens of yen worth of points that can be used as gift certificates or donations were given to all participants upon completion of the study by the survey company.

The study was registered as a University Hospital Medical Information Network Clinical Trials Registry Clinical Trial (Unique Trial Number: UMIN000045387) on 5 September 2021. The methods of the present study adhered to CONSORT guidelines [32]. The protocol was approved by the ethical review committee at the Graduate School of Medicine, University of Tokyo (number 2021155NI). All participants gave written informed consent in accordance with the Declaration of Helsinki.

### 2.3. Sample Size

We calculated the sample size during the study design phase. Because no study using an evolutionary theoretical approach examined the influence on health decision-making, based on a summary of effect sizes of social influence research in social psychology over a century [33], we estimated a small effect size (f  =  0.10) in the present study. We conducted a power analysis at an alpha error rate of 0.05 (two-tailed) and a beta error rate of 0.20. The power analysis using G * Power 3 [34] indicated that 323 participants were required in each of the intervention and control groups.

### 2.4. Intervention and Control Messages

Appendix A shows the intervention and control messages, translated into English for this report. We created intervention messages that targeted the fundamental motive of kin care and that targeted the fundamental motive of disease avoidance by referring to information from the Ministry of Health, Labor and Welfare. As mentioned earlier, existing HPV vaccination recommendation messages have primarily communicated content of disease avoidance. We created the intervention disease avoidance message that mimics typical existing HPV vaccination recommendation messages in Japan (e.g., see a website [35]).

Participants in the intervention group that received a message targeting the fundamental motive of kin care read a short comment and viewed a short video about parenting, just prior to reading the intervention message. This comment and video were used to activate the fundamental motive of kin care.

In this study, in order to detect the effect of the message using an evolutionary approach on HPV vaccination by comparing the intervention and control groups, the control message had to be content that did not affect the outcome, i.e., content that was not related to HPV vaccination. Therefore, for a control message we obtained textual information about bruxism from the website of the Ministry of Health, Labour and Welfare (https://www.e-healthnet.mhlw.go.jp/, last accessed on 14 February 2022 ).

Before reading the intervention or control message, participants were presented with the following instruction. “Please read the following pages carefully. Otherwise, you will not be able to answer the questions that follow. The next page will be fixed for 10 s (you will not be able to move on until 10 s have passed)”.

### 2.5. Measures

The primary outcome was intention to have daughter(s) receive the HPV vaccine. Participants responded to the following three questions on a scale of 1 to 6, ranging from “extremely unlikely” to “unlikely”, “a little unlikely”, “a little likely”, “likely”, and “extremely likely”: (1) If I could make an appointment right now to get the HPV vaccines, I would do it right now so that I could get my own daughter vaccinated. (2) Even if I am busy, I will make time to get my daughter the HPV vaccines. (3) Even if I am worried about the side effects of the HPV vaccines, I will get my daughter vaccinated. We adapted this measure from previous studies and modified it for the present study [36,37].

The secondary outcome was attitude toward HPV vaccination. Participants rated “having my daughter(s) receive the HPV vaccine” on a scale consisting of six 1–6 semantic differential items (bad/not bad, beneficial/not beneficial, harmful/not harmful, good/not good, valuable/not valuable, and important/not important). We adapted this measure from previous studies and modified it for the present study [38,39].

Participants also responded to the following question on a scale of 1 to 6 as mentioned above, “Doing something for my daughter’s child (my grandchild) in the future is my greatest motive among my various motives”. This question served as a manipulation check to examine if the intervention message that targeted the fundamental motive of kin care activated this fundamental motive of the participants.

All these questions were measured before and immediately after the participants read intervention or control messages. Mean scores (ranged from 1 to 6) were used for the analysis. Higher scores indicate greater intentions, more favorable attitudes, and greater motives. Additionally, all participants were asked for their sociodemographic information before they read intervention or control messages.

### 2.6. Statistical Analysis

Descriptive statistics were used to summarize participants’ sociodemographic information as percentages for categorical variables and as mean ± standard deviation for continuous variables. Cronbach’s α values were used to determine the internal reliability of the outcome measures. A one-way analysis of variance (ANOVA) was conducted with the absolute change in mean values before and after intervention and the mean values after intervention for each measure as the dependent variable and the group assignment as the independent variable. For multiple comparisons, Tukey’s test was conducted on significant main effects where appropriate. The Games–Howell test was performed when the assumption of homogeneity of variances was not satisfied. A *p* value of <0.05 was considered significant in all statistical tests. All statistical analyses were performed using IBM SPSS Statistics for Windows, Version 21.0 (IBM, Armonk, NY, USA).

## 3. Results

### 3.1. Participant Characteristics

Table 1 shows the participants’ characteristics. Participant age ranged from 30 to 59 years (mean = 45 years, standard deviation (SD) = 4.7). A total of 34% of participants had an educational attainment level beyond university graduation. A total of 53% of participants had an annual household income of six million yen or more (one US dollar is roughly equivalent to 100 yen). Participants were distributed throughout Japan.

### 3.2. Comparison of Outcomes between Groups

Cronbach’s α for internal consistencies of questions were 0.919 in intention to have daughter(s) receive HPV vaccination and 0.935 in attitude toward having daughter(s) receive HPV vaccination.

Table 2 shows the intention of receiving and attitude toward HPV vaccination across groups. Regarding the absolute change in mean values for intention of vaccination before and after the intervention, ANOVA revealed a significant main effect of the group assignment [*F*(2, 966) = 54.281, *p* < 0.001, η^2^ = 0.101]. The Games–Howell post hoc test revealed significant differences between the kin care and the control groups (M = 0.69 vs. M = 0.14, *p* < 0.001), and the disease avoidance and the control groups (M = 0.64 vs. M = 0.14, *p* < 0.001). There was no significant difference between the two intervention groups. Regarding the mean values for intention of vaccination after the intervention, ANOVA and multiple comparisons revealed the same trend as the absolute change.

Regarding the absolute change in mean values for attitude toward vaccination before and after the intervention, ANOVA revealed a significant main effect of the group assignment [*F*(2, 966) = 23.457, *p* < 0.001, η^2^ = 0.046]. The Games–Howell post hoc test revealed significant differences between the kin care and the control groups (M = 0.38 vs. M = 0.08, *p* < 0.001), and the disease avoidance and the control groups (M = 0.30 vs. M = 0.08, *p* < 0.001). There was no significant difference between the two intervention groups. Regarding the mean values for intention of vaccination after the intervention, ANOVA and multiple comparisons revealed the same trend as the absolute change.

### 3.3. Manipulation Check

As Table 2 shows, for absolute change in mean values for the motive of kin care before and after the intervention, ANOVA revealed a significant main effect of the group assignment [*F*(2, 966) = 5.034, *p* = 0.007]. The motive of kin care increased the most in the intervention group that targeted the fundamental motive of kin care; the Games–Howell post hoc test revealed a marginally significant difference between the kin care and the disease avoidance groups (M = 0.35 vs. M = 0.22, *p* = 0.054) and revealed a significant difference between the kin care and the control groups (M = 0.35 vs. M = 0.18, *p* = 0.001).

## 4. Discussion

We conducted interventions to examine the effectiveness of an HPV vaccine recommendation message that targeted the fundamental human motive of kin care of mothers with daughter(s), based on the evolutionary theoretical approach. Our hypothesis was supported by the study results: an HPV vaccination recommendation message that targeted the fundamental motive of kin care was as effective in encouraging HPV vaccination as a message that targeted the fundamental motive of disease avoidance. Our results indicate that previous strategies for communicating vaccination recommendations, which primarily used messages that targeted the fundamental motive of disease avoidance, was not the only optimal choice. It is recommended that health professionals add messages that target the fundamental motive of kin care to their repertoire of HPV vaccination recommendations.

A message that targeted the fundamental motive of kin care significantly improved mothers’ attitudes toward HPV vaccination and significantly increased mothers’ intention to have their daughter(s) receive the HPV vaccine compared to a control message. Previous research and practice on behavior change, including vaccination recommendations, have been based mainly on cognitive behavioral models, and focused on cognitive beliefs such as perceived susceptibility and severity of infection [9,10]. Previous studies have shown that many vaccine recommendation messages target the fundamental motive of disease avoidance and communicate the causes of infectious diseases and the benefits of vaccines (e.g., Let’s get vaccinated because vaccines prevent infection) [25,26,30]. However, repeated exposure to messages with similar themes generates a psychological reactance and disengagement toward incoming messages, leading to ineffective persuasive outcomes [28,29]. Therefore, the results of this study imply that health professionals should deliver messages that target not only the fundamental motive of disease avoidance, but also the fundamental motive of kin care, to increase the effectiveness of communication to encourage HPV vaccination.

This communication strategy targeting the fundamental motive of kin care may bring a new option to health professionals who experience complexity and difficulty in communicating about the HPV vaccine [30]. First, low health literacy levels are associated with low acceptance of the HPV vaccine [40]. General vaccine information requires a certain level of literacy and numeracy to understand, making it difficult for those with low health literacy to understand and use vaccine information [41,42]. However, messages that target the fundamental motive of kin care are simple and straightforward and may influence people of all health literacy levels. Second, health organizations and professionals are recommended to deliver effective tailored messages for addressing vaccine hesitancy [43,44]. Messages that target the fundamental motive of kin care can be used to deliver tailored personally relevant messages, which is recommended to amplify vaccine affirmation [45]. Third, messages that target the fundamental motive of kin care can be used in multiple channels—which are also recommended for effective vaccine communication—such as online, offline and face-to-face communication [44,46,47]. For example, provision of HPV vaccine information by trusted health care providers, combined with the provision of influential HPV vaccine information, can increase confidence to receive HPV vaccination [48,49,50]. Messages that target the fundamental motive of kin care can be used in such integrated HPV vaccine communication approach by embedding the message in various channels.

A message that targeted the fundamental motive of disease avoidance also significantly improved mothers’ attitudes toward HPV vaccination and significantly increased mothers’ intention to have their daughter(s) receive the HPV vaccine compared to a control message. Vaccine recommendation messages to date have mainly targeted the fundamental motive of disease avoidance [25,26,30]; the results of this study indicate that these vaccine recommendation messages have had some effect in encouraging HPV vaccination. Therefore, the results of this study imply that health professionals continue to deliver messages that target the fundamental motive of disease avoidance to encourage HPV vaccination (e.g., the ease of infection, the severity of symptoms if infected, and the effectiveness of vaccines).

The present study found no significant difference in effectiveness between the two intervention messages; namely, a message that targeted the fundamental motive of kin care was as effective as a message that targeted the fundamental motive of disease avoidance to encourage mothers to have their daughter(s) receive the HPV vaccine. As mentioned earlier, we created the intervention disease avoidance message that mimics typical existing HPV vaccination recommendation messages in Japan. Therefore, the result indicates that the kin care message was as effective as existing messages. As noted above, using messages that target the fundamental motive of kin care will extend the communication strategy for HPV vaccination recommendations.

Future studies should examine whether a combination message (i.e., kin care plus disease avoidance) is more effective than a kin care only message or a disease avoidance only message. Future studies should also examine the effect of messages that target the fundamental motive of kin care on encouraging health behavior other than HPV vaccination. For example, messages that target the fundamental motive of kin care may encourage women who wish for future childbirth and parenting to undergo cervical cancer screening. Additionally, there are other fundamental human motives than kin care, such as affiliation, status, mate acquisition, and mate retention. Future studies should examine the effectiveness of public health messages including vaccination recommendations that target these fundamental motives. It will be useful to conduct qualitative studies to get a deeper understanding of facilitators and barriers regarding HPV vaccination among parents in Japan in terms of evolutionary theoretical approach.

The present study has several limitations. First, although participants were instructed to read messages carefully before reading the intervention or control message, it is unclear to what extent the participants perused the messages. Second, because the content of the control message was not associated with HPV vaccination, it cannot be determined that the intervention messages targeting the fundamental motives encouraged vaccination. For example, the results of this study may have just detected an effect of HPV vaccination information. However, we created the intervention disease avoidance message that mimics typical existing HPV vaccination recommendation messages in Japan. Therefore, the results indicate that the kin care message was as effective as existing messages, and they have important implications to extend strategies of HPV vaccination communication as mentioned earlier. Third, this study assessed vaccination intentions directly after message exposure. Our previous study showed that HPV vaccination intention 4 months after intervention using statistics, a patient’s and a mother’s narrative had decreased to a level that did not differ significantly from the level prior to intervention [51]. Future studies should examine the long-term effects of messages using an evolutionary approach because they are important in this context given that HPV vaccination requires multiple injections over a series of weeks. Fourth, this study assessed vaccination intentions rather than actual vaccination behaviors; future studies should examine the effects of messages on actual vaccination behaviors. Fifth, the possibility of spillover effects between groups cannot be completely ruled out; although since this is an internet survey and not a study conducted in one limited site or region, the possibility of spillover effects is considered small. Finally, it is unclear to what extent the present findings are generalizable to populations other than the Japanese participants in this web-based survey.

## 5. Conclusions

This is the first study to examine the effectiveness of HPV vaccination recommendation messages by focusing on the fundamental human motive based on the evolutionary theoretical approach. We found that a message that targeted the fundamental human motive of kin care—which has been rarely used—was as effective in encouraging HPV vaccination among mothers with daughter(s) as a message that targeted the fundamental human motive of disease avoidance, which has been frequently used in cognitive behavioral models. This result indicates that the evolutionary theoretical approach that focuses on fundamental human motives has the potential to extend the communication strategy for HPV vaccination recommendations. Health professionals should deliver messages that target the fundamental motive of kin care, in addition to messages about susceptibility and severity of HPV infection and HPV vaccine efficacy, to encourage HPV vaccination among parents with daughter(s). Messages that target the fundamental motive of kin care are those such as, “Getting cervical cancer can prevent childbirth. To protect your daughter and your future grandchildren, get your daughter vaccinated against HPV”.

## Figures and Tables

**Table 1:** Participants’ characteristics.

	Kin Care (n = 323)	Disease Avoidance (n = 323)	Control (n = 323)	Total (n = 969)
Age, mean years (SD)	44.7 (4.4)	44.6 (5.2)	45.1 (4.6)	44.8 (4.7)
Highest education, %				
Less than high school	3.1	0.9	1.9	2.0
High school graduate	23.8	26.3	25.7	25.3
Some college	38.7	38.7	39.6	39.0
University graduate	30.3	31.9	29.1	30.4
Graduate school	4.0	2.2	3.7	3.3
Household income, %				
Less than 2 million yen ^a^	6.8	5.3	5.6	5.9
2–4 million yen	17.3	17.0	16.7	17.0
4–6 million yen	24.8	22.9	26.0	24.6
6–8 million yen	24.1	25.4	19.8	23.1
8–10 million yen	13.9	16.1	14.9	15.0
More than 10 million yen	13.0	13.3	17.0	14.4

^a^ One US dollar is roughly equivalent to 100 yen.

**Table 2:** Comparisons of measures between groups.

		Kin Care (n = 323)	Disease Avoidance (n = 323)	Control (n = 323)	*p * ^d^
Intention of vaccination	Before	2.85 ^a^ (1.32) ^b^	2.84 (1.22)	2.85 (1.24)	N/A
	After	3.53 (1.30) **	3.49 (1.24) **	2.99 (1.28)	<0.001
	Change	0.69 (0.60–0.78) ^c^ **	0.64 (0.56–0.72) **	0.14 (0.07–0.21)	<0.001
Attitude toward vaccination	Before	3.73 (1.17)	3.72 (1.06)	3.68 (0.99)	N/A
	After	4.11 (1.14) **	4.02 (1.08) *	3.76 (1.05)	<0.001
	Change	0.38 (0.31–0.45) **	0.30 (0.23–0.36) **	0.08 (0.04–0.13)	<0.001
Motive of kin care	Before	3.98 (1.32)	3.99 (1.40)	3.95 (1.44)	N/A
	After	4.33 (1.31)	4.20 (1.40)	4.13 (1.40)	0.180
	Change	0.35 (0.26–0.43) *	0.22 (0.14–0.29)	0.18 (0.10–0.25)	0.007

^a^ Mean. ^b^ Standard deviation. ^c^ 95% confidence interval. ^d^ *p* values for comparing amount of change among groups using ANOVA. * Significantly higher than the control group by multiple comparisons (*p* = 0.05). ** Significantly higher than the control group by multiple comparisons (*p* < 0.001). N/A: Not applicable.

## Data Availability

The datasets generated and/or analysed during the current study are available from the corresponding author on reasonable request.

## Appendix A. Intervention and Control Messages Used in This Study, Translated into English

**An intervention message that targeted the fundamental motive of kin care**
Cervical cancer is caused by a virus that is transmitted through sexual intercourse.
Cervical cancer is the most common cancer in women in their 20s and 30s.
Cervical cancer has almost no early symptoms.

If cervical cancer is detected late, it may result in surgery to remove the uterus, making it impossible to conceive and give birth.
Even if the patient can conceive, there is an increased chance of premature birth or low birth weight babies.

Cervical cancer can be prevented by getting vaccinated against the disease.
Severe side effects rarely persist, with 2 cases per 100,000 vaccinations (about 0.002%).

Girls in the equivalent of 6th grade to 1st grade of high school are eligible for vaccination.
For the sake of your daughter’s future childbirth parenting, have your daughter vaccinated against cervical cancer.

**An intervention message that targeted the fundamental motive of disease avoidance**
Cervical cancer is caused by a virus that is transmitted through sexual intercourse.
Cervical cancer is the most common cancer in women in their 20s and 30s.
Cervical cancer has almost no early symptoms.

If cervical cancer is detected late, four out of five women will die.
Even if it can be treated, there may be after-effects such as difficulty in defecating and urinating.

Cervical cancer can be prevented by getting vaccinated against the disease.
Severe side effects rarely persist, with 2 cases per 100,000 vaccinations (about 0.002%).

Girls in the equivalent of 6th grade to 1st grade of high school are eligible for vaccination.
Have your daughter vaccinated against cervical cancer.

**A control message**
According to the traditional definition, grinding one’s teeth is when somebody makes a sound by strongly grinding the teeth together, usually unconsciously or while asleep. Nowadays, it is often referred to as ‘teeth grinding,’ a term which also covers various actions that we do whilst awake.

Whether you are sleeping or awake, the non-functional biting habit of grinding one’s teeth dynamically or statically, or clenching one’s teeth, can also be referred to as bruxism (sleep bruxism if it occurs at night). Bruxism can be categorized into the movements of: sliding the upper and lower teeth together like mortar and pestle (grinding); firmly and statically engaging the upper and lower teeth (clenching); and dynamically bringing the upper and lower teeth together with a tap (tapping).

Bruxism is difficult to diagnose, as it often has no noticeable symptoms. Stress and dentition are thought to be causes of bruxism, but it is currently unclear and future research is anticipated.
